# On the frequency dependence of viscoelastic material characterization with intermittent-contact dynamic atomic force microscopy: avoiding mischaracterization across large frequency ranges

**DOI:** 10.3762/bjnano.11.125

**Published:** 2020-09-15

**Authors:** Enrique A López-Guerra, Santiago D Solares

**Affiliations:** 1 The George Washington University, Department of Mechanical and Aerospace Engineering, Washington, DC 20052, USA; 2 Park Systems Inc., Santa Clara, CA, 95054, USA

**Keywords:** dynamic atomic force microscopy, Generalized Maxwell model, loss modulus, storage modulus, viscoelasticity

## Abstract

Atomic force microscopy (AFM) is a widely use technique to acquire topographical, mechanical, or electromagnetic properties of surfaces, as well as to induce surface modifications at the micrometer and nanometer scale. Viscoelastic materials, examples of which include many polymers and biological materials, are an important class of systems, the mechanical response of which depends on the rate of application of the stresses imparted by the AFM tip. The mechanical response of these materials thus depends strongly on the frequency at which the characterization is performed, so much so that important aspects of behavior may be missed if one chooses an arbitrary characterization frequency regardless of the materials properties. In this paper we present a linear viscoelastic analysis of intermittent-contact, nearly resonant dynamic AFM characterization of such materials, considering the possibility of multiple characteristic times. We describe some of the intricacies observed in their mechanical response and alert the reader about situations where mischaracterization may occur as a result of probing the material at frequency ranges or with probes that preclude observation of its viscoelastic behavior. While we do not offer a solution to the formidable problem of inverting the frequency-dependent viscoelastic behavior of a material from dynamic AFM observables, we suggest that a partial solution is offered by recently developed quasi-static force–distance characterization techniques, which incorporate viscoelastic models with multiple characteristic times and can help inform dynamic AFM characterization.

## Introduction

There have been significant methodology developments since the introduction of atomic force microscopy (AFM) in the mid-1980s [1–3]. Besides its extensive use for topographical measurements, AFM is employed routinely for mechanical characterization [4–14], among other applications. Within mechanical characterization, materials may be broadly classified as elastic or viscoelastic, not considering situations where permanent and abrupt structural changes take place, such as those observed in plastic processes. Elastic materials are those for which the force–distance curve does not exhibit hysteresis, such that the work done by the probe on the material during the approach portion of the curve is recovered by the probe upon retraction, with the probe–sample forces being independent of the rate of deformation (in the ideal case) [15–16]. In contrast, during the characterization of viscoelastic materials, hysteresis is observed in the force–distance curve, as the material yields and does not fully recover its original geometry upon retraction of the probe [15–18]. The probe does not recover all the work it originally imparted to the material, and the material remains deformed and gradually approaches its original geometry according to its characteristic relaxation timescales, although it may or may not fully recover its original geometry. Prime examples of such materials include polymers and biological materials [2,14,19–21].

The peculiar types of behavior of viscoelastic materials have been recognized by AFM researchers since the early days of dynamic AFM, and various studies have explored different phenomena, such as energy dissipation and the proportion between elastic and viscous probe–sample interactions during the measurement [22–27]. A large number of publications have focused on the study of the phase signal (i.e., the lag of the oscillatory response of the cantilever with respect to the excitation, within amplitude-modulation AFM (AM-AFM)), which generally yields high-contrast images for dissipative materials [22]. Dynamic contact-mode techniques such as contact-resonance AFM [11–13,28], dual-amplitude resonance tracking AFM (DART [10]) and band-excitation AFM [9,29], as well as dynamic methods based on multifrequency AFM [4–5] and multi-harmonic AFM [30–31] have also been implemented to measure an effective modulus of elasticity and an effective coefficient of dissipation (or analogous quantities) across the surface. All of these methods have found niches of application and have provided physical insight into a wide variety of materials.

**Figure 1:**
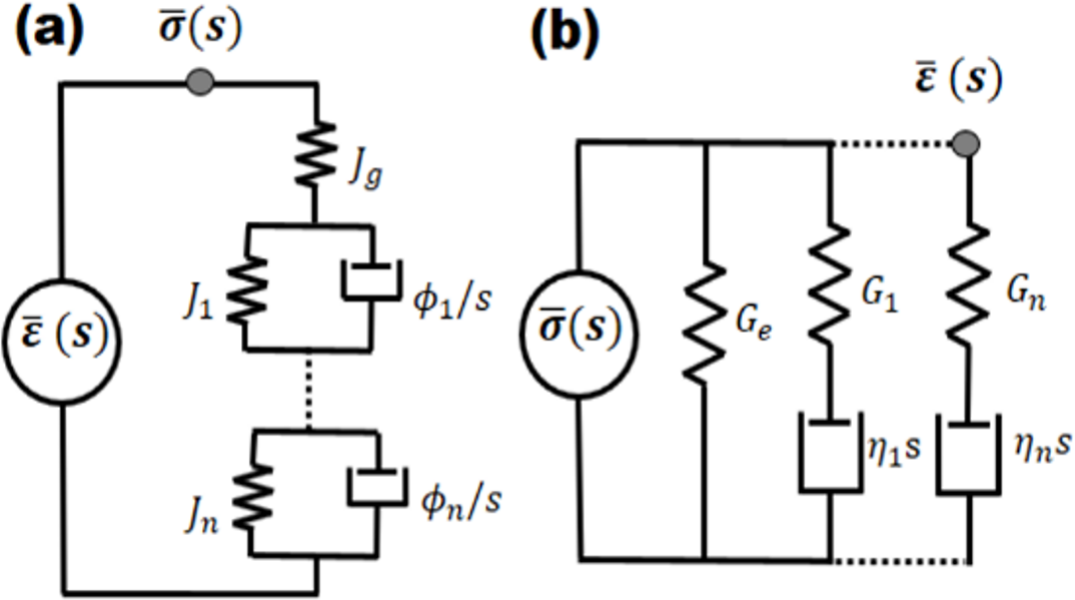
Mechanical model diagrams representing the relationship between stress and strain in the complex plane for a linear viscoelastic material with multiple characteristic times [33]. (a) Generalized Voigt or Kelvin model, (b) Generalized Maxwell or Wiechert model, both describing arrheodictic behavior (i.e., without steady-state flow). *J_n_* and *G_n_* refer to the compliance and the modulus of the *n*-th spring, respectively. ϕ*_n_* and η*_n_* refer to the fluidity and viscosity of the *n*-th damper, respectively. *J*_g_ and *G*_e_ refer to the glassy compliance and the rubbery modulus, respectively. In (a) the Laplace transformed strain 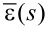 is generally regarded as the excitation and the transformed stress 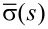 as the response; in (b) the opposite generally occurs.

In keeping with the above trajectory in the development of new AFM methods for viscoelastic materials, in recent years we have focused on the application of linear viscoelasticity within quasi-static force spectroscopy (i.e., using force–distance curves acquired at frequencies much lower than the resonance frequency of the cantilever). Specifically, we have introduced a methodology based on the Generalized Voigt (Kelvin) or Maxwell viscoelastic model (Figure 1), including an arbitrary number of characteristic viscoelastic timescales, and on a previous mathematical development by Lee and Radok for spherical indenters deforming flat incompressible surfaces [32]. Lee and Radok’s expression for the Laplace transformed indentation depth, *h*, is

[1]
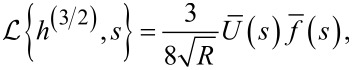


where, 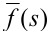 is the transformed load (applied force), 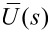 is the retardance of the material, relating stress and strain, and *R* is the indenter radius. The indentation and load are available from the force–distance curve, and an expression for 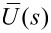 can be easily derived for the Generalized Voigt or Maxwell models (Figure 1), whereby the constants of springs and dampers of the model are initially left unspecified. Property inversion is then performed through a nonlinear regression procedure that yields the constants of the springs and dampers [14,33]. Note that for this analysis one considers only the repulsive-contact region. This approach has been applied to synthetic polymer samples and biological materials. For example, we have recently applied it to map the mechanical properties of biofilms and single cells, describing their behavior with respect to time and frequency [14,33]. A similar approach to ours, which also profits from the elastic–viscoelastic correspondence principle, was introduced by Efremov et al. [34], which they applied to living cells. In contrast to the discrete nature of the Generalized Maxwell model, which allows the use of arbitrary parameters at different time scales modeled by different spring–damper combinations, they focused primarily on the standard-linear-solid model and on power-law rheological models, which can be thought of as infinite collections of spring–damper combinations that form a continuous relaxation spectrum governed by a power-law. In both approaches, from the constants of the model one can easily obtain the viscoelastic harmonic functions as a function of frequency in the range of frequencies involved in the experiment. For example, for the Generalized Maxwell model, which this paper focuses on, the storage shear modulus, *G*′, which accounts for the elastic behavior of the material under harmonic excitation, is given by [15]:

[2] $$G^{\prime }\left(\omega \right)=G_{\text{e}}+\sum_{i=1}^{n}G_{i}\left(1-\frac{1}{1+\omega^{2}\tau_{i}^{2}}\right),$$

where ω is the angular frequency, equal to 2πν. At zero frequency, *G*′, is equal to *G*_e_, the rubbery shear modulus, and as the frequency increases, it converges to the glassy shear modulus, *G*_g_, which is given by:

[3] $$G_{\text{g}}=G_{\text{e}}+\sum_{i=1}^{n}G_{i}.$$

The loss shear modulus, *G*″, which accounts for the viscous behavior of the material, is given by [15]:

[4] $$G^{\prime \prime }\left(\omega \right)=\sum_{i=1}^{n}\frac{G_{i}\omega \tau_{i}}{1+\omega^{2}\tau_{i}^{2}},$$

where G″(ω) at both zero and infinitely large frequencies converges to zero, implying pure elastic behavior at those extrema. Note that the above equations and paragraphs refer to shear moduli (e.g., storage shear modulus and loss shear modulus) instead of tensile (Young´s) moduli, in order to follow the convention often used in linear viscoelasticity theory, which is related to the fact that the moduli directly measured in many classical experiments (e.g., using rheometers) are shear moduli. However, the relationship between the viscoelastic shear modulus (*G*) and the viscoelastic Young´s modulus (*E*) is straightforward when the Poisson’s ratio (ν) is considered time-independent:

[5] 2G(1+ν)=E.

In the remainder of the manuscript we omit the designation ‘shear’ for simplicity, although in all cases we refer to shear moduli.

**Figure 2:**
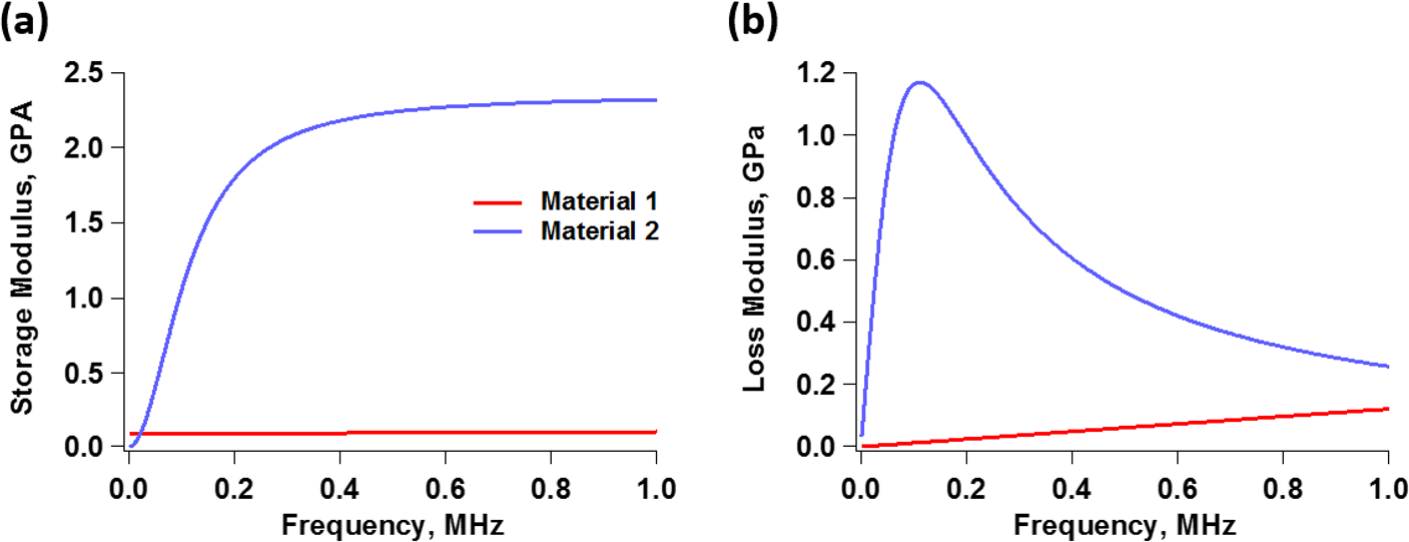
(a) Storage moduli and (b) and loss moduli as function of frequency for two hypothetical materials, the Generalized Maxwell model parameters of which are provided in Table 1. The plots include only the frequency range that is relevant to the simulations reported in this study. The plots in (a) correspond to Equation 2 while the plots in (b) correspond to Equation 4.

Figure 2 illustrates loss and storage modulus as function of the frequency of two hypothetical materials, the Generalized Maxwell model parameters of which are provided in Table 1. It is clear from the graphs that both the storage and the loss modulus can vary significantly as a function of the deformation frequency, which has very important implications in the context of dynamic force spectroscopy. First, for samples exhibiting such variation in their moduli, it is not possible to assign a single value to the coefficient of viscous dissipation during deformation, since the loss modulus is not constant. The variation in the elastic modulus also precludes the definition of a single elastic modulus for the material. Furthermore, we see that neither the loss nor the storage modulus exhibit trivial behavior for the examples shown. For example, depending on the frequency range considered, the loss modulus may or may not exhibit monotonic variation, and the slopes of the moduli with respect to frequency can vary widely.

**Table 1:** Maxwell model parameters for the materials simulated in this study. The characteristic time (τ_i_) associated with each spring–damper combination (“arm”) of the model is given by the ratio of the viscosity of the damper in the *i*-th arm (η_i_ ) to the modulus of the spring in the *i*-th arm (*G_i_*): τ*_i_* = η*_i_*/*G_i_*.

	material 1	material 2	material 3

*G*_e_, Pa	1 × 10^8^	1 × 10^7^	1 × 10^7^
*G*_1_, Pa	1.5 × 10^8^	2.34 × 10^9^	2.34 × 10^9^
*G*_2_, Pa	1.1 × 10^9^	—	2 × 10^9^
*G*_3_, Pa	1 × 10^9^	—	—
*G*_g_ (glassy modulus), Pa	2.35 × 10^9^	2.35 × 10^9^	2.35 × 10^9^
η_1_, Pa·s	0.428	3.342 × 10^3^	3.342 × 10^3^
η_2_, Pa·s	4.715	—	60
η_3_, Pa·s	14.29	—	—
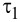 , s	2.857 × 10^−9^	1.429 × 10^−6^	1.429 × 10^−6^
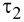 , s	4.286 × 10^−9^	—	3 × 10^−8^
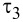 , s	1.429 × 10^−8^	—	—

The behavior of storage and loss moduli as a function of frequency can be easily understood by inspection of the models of Figure 1. Consider the Generalized Maxwell model (Figure 1b). At very low frequencies, the deformation rate is too slow for any of the dampers (η*_i_*) to generate any resistance, so they all yield without dissipating energy. Thus, the material behaves as elastic, with a modulus equal to the rubbery modulus *G*_e_, since *G*_e_ is the only element that is able to act at such low frequencies. This is known as the rubbery regime, which is characterized by a soft-elastic behavior [15,18]. At the other end of the spectrum, at very high frequencies, the deformation rate is extremely high, so the force required for any of the dampers to yield is too high. Therefore, the dampers remain “locked” in position, and all the arms of the model behave as springs. In this case, there is no dissipation at the dampers (since they are not yielding) and the material behaves as elastic, with a modulus equal to the glassy modulus *G*_g_, which is the sum of the moduli of all springs in the model. This is known as the glassy regime, which is characterized by a stiff-elastic behavior [15,18]. Since both of these extreme types of behavior are elastic, the loss modulus is zero at zero frequency and also decreases, asymptotically approaching zero, at very high frequencies. At intermediate frequencies, depending on their characteristic time, one or more dampers may require only a moderate force to yield, so they do yield, dissipating energy according to the force they sustain and the velocity at which they are being deformed. This is why the loss modulus peaks for intermediate values of the frequency. (Note that in Figure 2b the loss modulus of material 1 has not yet peaked in the range of frequency shown in the graph – a similar plot over a wider frequency range is discussed in the Results and Discussion section).

Although all of the above phenomena occur similarly within intermittent-contact dynamic AFM methods (such as tapping-mode AFM), the analysis of the sample properties is not as simple as for quasi-static force curves, and Equation 1 cannot be directly applied. The force is not generally known as a function of the tip position in dynamic AFM, and the material may not fully recover its original geometry between subsequent tip impacts [35]. Inversion of the dynamic AFM observables to obtain a sample model similar to the models shown in Figure 1 is beyond the scope of this paper, as this is a much more difficult problem than the analogous problem for quasi-static AFM [14,33–34]. Nevertheless, we believe there is significant value in analyzing (through simulation) the behavior of the dynamic AFM observables for hypothetical materials as a function of the frequency. We find this to be particularly important, as a review of the literature (including several of our own previous works), shows that dynamic AFM characterization has been routinely performed with different types of cantilevers (short high-frequency cantilevers as well as traditional cantilevers) and with different methods, involving wide frequency ranges and different cantilever eigenmodes. In general, this is routinely done without considering the types of viscoelastic behavior described above, in particular the variation of the storage and loss moduli with frequency. In the remainder of this manuscript we present a short analysis of simulated types of behavior in phase and amplitude spectroscopy curves, as well as force–distance trajectories, with particular focus on the challenges that arise in trying to discriminate between different materials exhibiting different characteristic times, when the latter are not considered in the analysis.

## Results and Discussion

**Figure 3:**
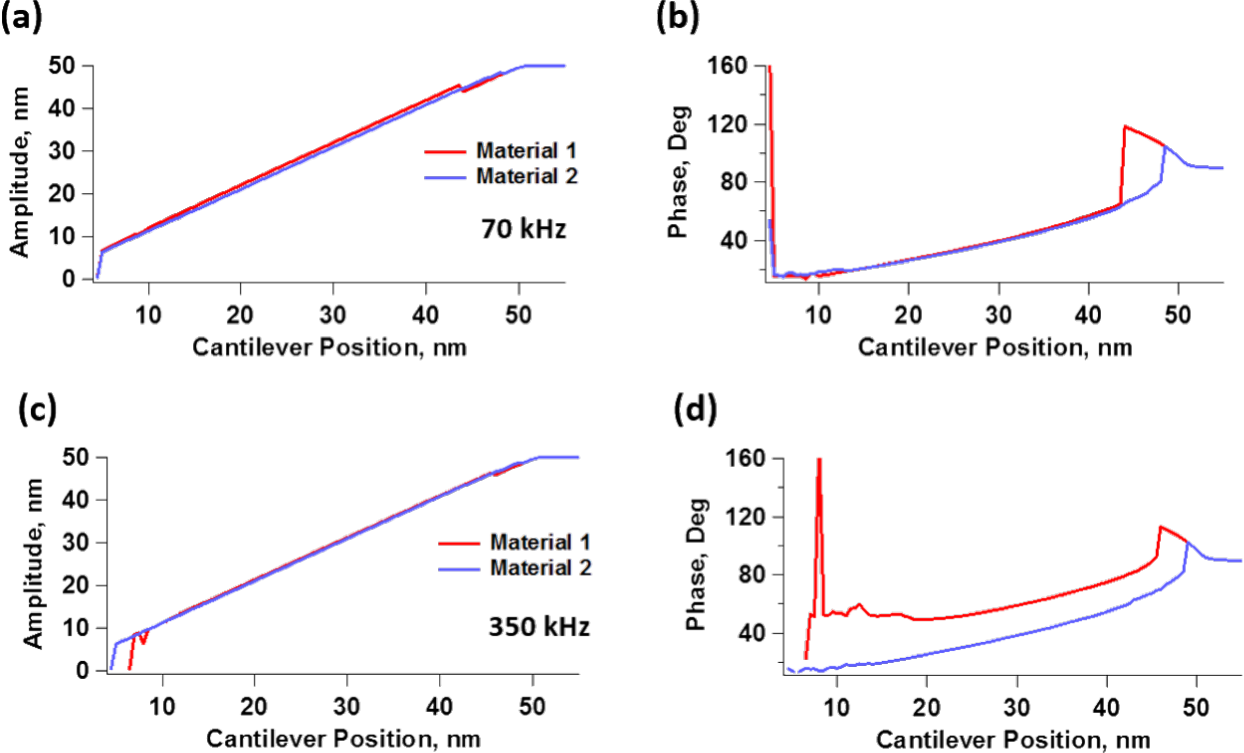
(Top row) Spectroscopy curves showing (a) amplitude and (b) phase as function of the cantilever position for constant drive amplitude and frequency, for a 70 kHz cantilever with a force constant of 0.5 N/m, using a free oscillation amplitude of 50 nm to characterize the two materials described in Figure 2 and Table 1. (Bottom row) Spectroscopy curves showing (c) amplitude and (d) phase as function of the cantilever position for constant drive amplitude and frequency, for a 350 kHz cantilever with a force constant of 0.5 N/m, using a free oscillation amplitude of 50 nm to characterize the abovementioned materials.

We first simulated the curves of amplitude and phase as function of the cantilever position for both materials described in Figure 2 and Table 1, when imaged with a soft tapping-mode cantilever. For this we used a resonance frequency of 70 kHz, a relatively low force constant of 0.5 N/m, considering the softness of the materials under study, and a free oscillation amplitude of 50 nm (the Experimental section below provides the simulation procedures and parameters). The amplitude curves (Figure 3a) exhibit the typical shape with bistability transitions [2]. We also notice that the amplitude values in the repulsive branch are larger for material 1 than for material 2. This is consistent with Figure 2, which shows that the moduli of material 1 are lower than those of material 2 in the vicinity of the characterization frequency, resulting in greater indentation for material 1. Note that both materials have the same glassy modulus (Table 1), and would thus behave equally stiff and elastic at very high frequencies. However, surprisingly, Figure 3b shows that both materials exhibit nearly the same phase behavior in the repulsive branch (the portion of the curve with phase values below 90°, dominated by repulsive tip–sample interactions), which is precisely the one that would be used to evaluate their viscoelastic mechanical properties. The attractive branch, which exhibits phase values above 90° is not relevant, as it is governed by attractive tip–sample interactions. The location of the sharp transitions between the attractive and repulsive oscillation regimes are the result of nonlinear dynamical behavior of the cantilever–sample system and are not directly related to materials properties [2]. Granted that spectroscopy methods are not based on phase alone, it is nevertheless interesting that both materials would appear similar according to phase contrast, such that a blend of both materials would be expected to exhibit very little to no phase contrast.

If we now consider imaging of the same materials using a cantilever with a larger resonance frequency of 350 kHz, but with the same low force constant of 0.5 N/m, we observe a very different result. Now the difference in indentation is smaller, as the amplitude curves of both materials are very similar to one another (Figure 3c), and the phase contrasts (Figure 3d) differ significantly, such that the phase image of a blend of the two materials would allow the user to immediately tell them apart under the chosen imaging conditions. (Note that the use of a cantilever with a resonance frequency of 350 kHz and a force constant of 0.5 N/m does not correspond to a realistic situation, as such high-frequency/low-stiffness cantilevers are not used in AFM. However, this example is included because it provides physical insight into the frequency dependence of viscoelastic properties).

**Figure 4:**
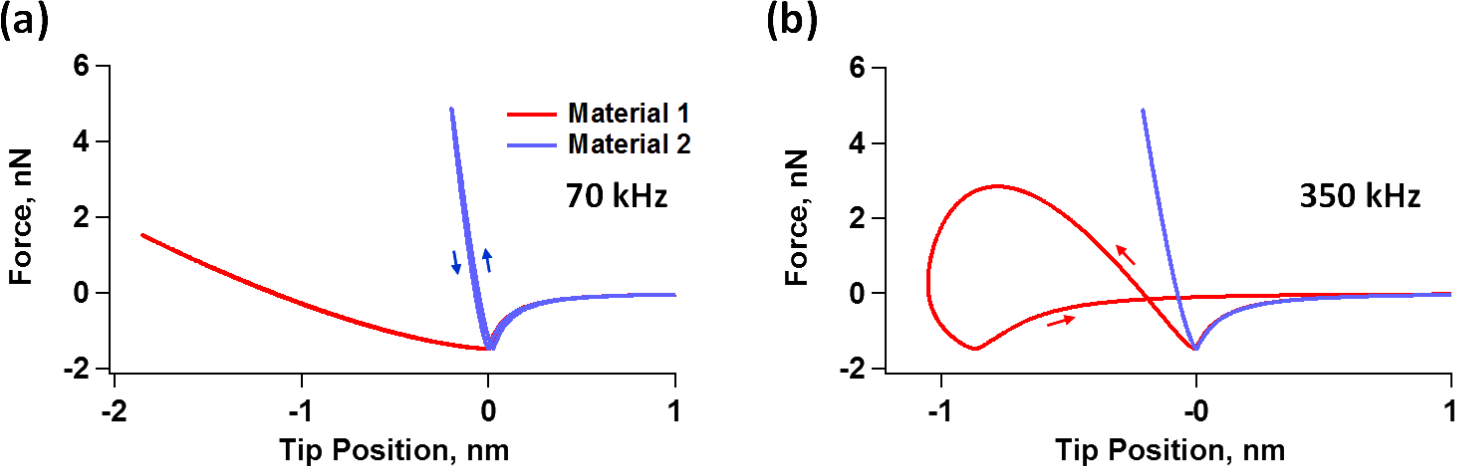
Force-vs-cantilever tip trajectory for characterization of the materials described in Figure 2 and Table 1 using a cantilever resonance frequency of (a) 70 kHz and (b) 350 kHz, for an amplitude setpoint of approximately 55%. The arrows indicate direction of the force trajectory as the tip travels towards and away from the sample. The hysteresis loops are the result of viscoelastic relaxation in the sample, whereby the distance between the two force minima on the curve can be approximately thought of as the distance that the surface has yielded (relaxed) due to its viscous behavior, while the area of the loops represents energy dissipation [35]. In (b) the surface of material 1 relaxes approximately 0.8 nm during the tip–sample impact such that the force trajectory the tip experiences during the approach differs significantly from the trajectory it experiences during the retract. The work performed by the tip on the sample during the approach is lower than the work performed by the sample on the tip during retract, causing the oscillating cantilever to experience a net loss in energy.

To obtain further insight, we now examine the force trajectories for the above simulations, which are shown in Figure 4 for an amplitude setpoint of approximately 55% (in the repulsive branch). Figure 4a confirms that the indentation is ca. 1.9 nm greater for material 1 than for material 2 at 70 kHz, whereby material 2 appears much stiffer than material 1. Furthermore, the near absence of dissipation in the force curves of both materials supports the observed lack of phase sensitivity to material differences, in agreement with the literature [36–37].

For the simulation at 350 kHz, Figure 4b again confirms the expectations, indicating that the difference in indentation between the two materials is smaller than at 70 kHz (notice that Figure 4a and Figure 4b have different horizontal scales). A calculation using Equation 2 indicates that the storage modulus for material 1 approximately doubles between 70 and 350 kHz, while a calculation with Equation 4 shows that its loss modulus increases by a factor of approximately 4.7 in the same frequency range. Additionally, material 1 now exhibits an obvious hysteresis loop, whereby the surface relaxes approximately 0.8 nm, leading to tip–sample dissipation and a different phase response between the two materials [36–37] (at 350 kHz material 2 shows an imperceptible hysteresis loop and similar indentation as for 70 kHz). The increase in hysteresis is also consistent with the increase in the loss modulus, evident in Figure 2b. A calculation using Equation 2 and Equation 4 shows that the storage and loss moduli of material 2 have increased by factors of approximately 4.9 and 3.7, respectively.

The above simulations illustrate the frequency dependence of the AFM observables for specific materials, along with the challenges involved in differentiating materials with fundamentally very different viscoelastic types of behavior on the basis of AM-AFM observables, namely amplitude and phase. While this may be unexpected, it should not be so if one considers that this imaging mode offers only two observables, which are the result of a larger number of intricacies in the materials, which can have multiple stiffness contributions and multiple relaxation timescales, as in the above examples, while the interplay and relative dominance of their viscoelastic contributions (e.g., the different arms in the Maxwell model) varies greatly with deformation frequency. This suggests that a full viscoelastic characterization of a material with AM-AFM would require procedures that are more elaborate than a simple two-dimensional scan, perhaps involving multiple volume scans using different cantilevers having different resonance frequencies. While we do not yet have a specific recommendation on what this procedure may look like, our results do suggest that the dimensionality and/or richness of the mechanical features we are seeking to characterize is appreciably greater than that of the available observables for traditional intermittent-contact modes (amplitude and phase in AM-AFM [2], and frequency and amplitude (or dissipation) in frequency-modulation AFM (FM-AFM [3]).

**Figure 5:**
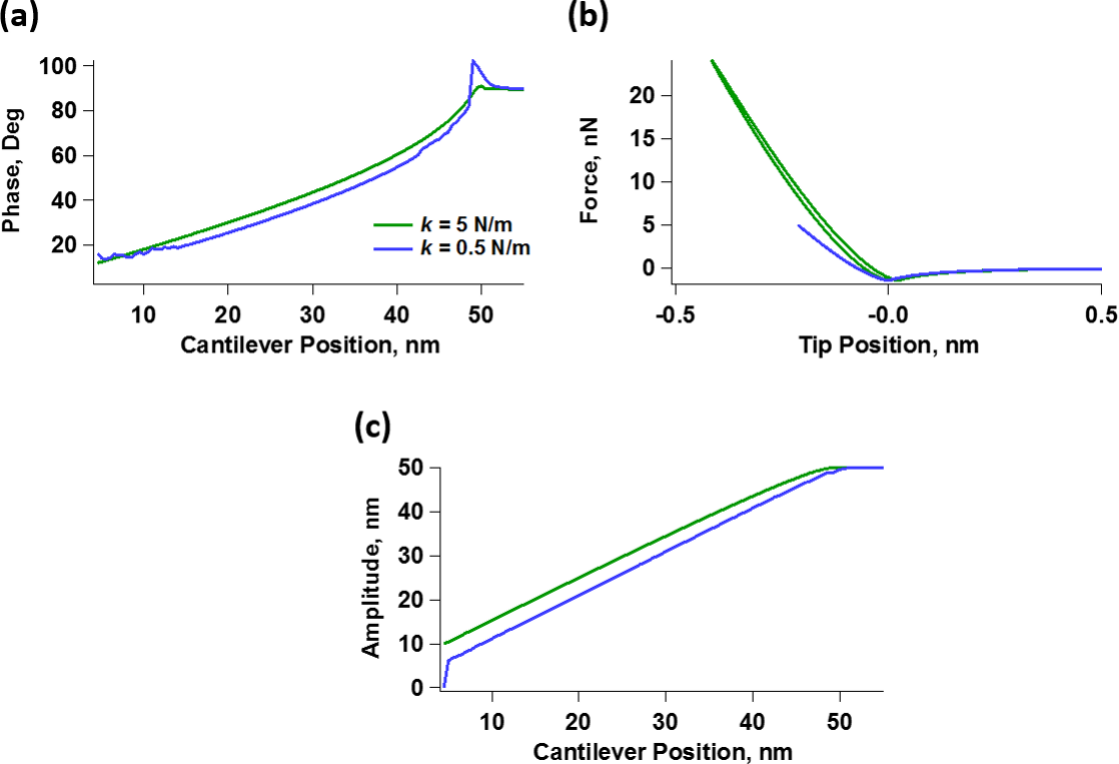
(a) Comparison of spectroscopy curves illustrating the phase as a function of the cantilever position for material 2 using the same parameters as for Figure 3d, which used a cantilever force constant of *k* = 0.5 N/m, and increasing the value of the cantilever force constant by an order of magnitude to *k* = 5 N/m. (b) Comparison of the corresponding force-vs-cantilever tip trajectories. (c) Comparison of the corresponding curves of the amplitude as a function of the cantilever position.

Another very important consideration concerning the ability to explore the full range of viscoelastic types of behavior in the sample is the ability of the cantilever to indent it. As the above results show, the indentation observed for material 2 in Figure 4 is much smaller than the indentation observed for material 1. This is not surprising considering that material 2 has much larger moduli than material 1, as shown in Figure 2, which may limit the ability of the low-*k* cantilevers used to properly characterize it. However, one should be surprised to see hardly any hysteresis in the force trajectory of material 2 despite its obvious viscoelastic nature depicted in Figure 2. One must therefore consider whether the cantilever stiffness used is appropriate, since the force constant has a direct impact on the ability of the cantilever to penetrate into the sample, when all other parameters remain constant [38]. Figure 5 shows simulation results for material 2 using a 350 kHz cantilever with the same parameters used to construct Figure 3c, Figure 3d and Figure 4b, but with a larger force constant of 5 N/m. Figure 5a compares the phase curves for the two cases (*k* = 0.5 N/m and *k* = 5 N/m). The plots show that the phase shift (the difference between 90° and the observed phase) is now smaller for both the repulsive and the attractive branches, indicating a greater dominance of the cantilever stiffness over the sample stiffness. Figure 5b compares the force trajectory during impact for both values of the force constant, and indeed, we observe significantly greater indentation and repulsive forces for the stiffer cantilever. Furthermore, the force trajectory now shows an obvious hysteresis loop, which the softer cantilever was not able to probe. Finally, we see that the oscillation amplitude as a function of the cantilever position is larger for the larger force constant, and also decreases less rapidly, confirming that the sample is less capable of perturbing the oscillation of the stiffer cantilever than the oscillation of the softer cantilever. Note that the ability of the cantilever to indent the sample also increases as the free oscillation amplitude is increased [38], so in some cases an increase in oscillation amplitude may be sufficient to more deeply probe the mechanics of the sample. Clearly the ability of the cantilever to sufficiently indent the sample must be considered in order to perform an accurate viscoelastic characterization of the material. A mathematical analysis of the variables that govern the indentation in dynamic AFM methods based on dimensional analysis of the equation of motion of the cantilever is provided in [38].

**Figure 6:**
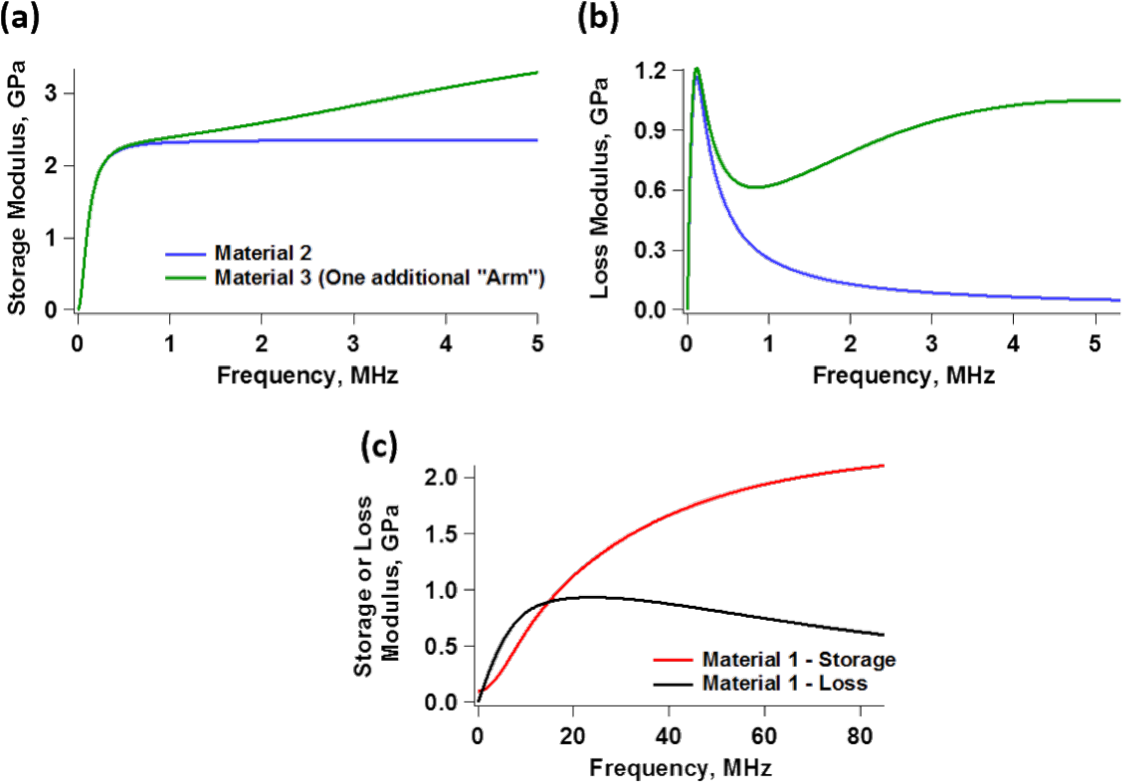
(a, b) Comparison of the storage and the loss modulus of material 2 (Figure 2a and Figure 2b, respectively) with a third material, which has the same viscoelastic elements of the Generalized Maxwell model used to represent material 2, but with an additional Maxwell arm having the parameters *G*_2_ = 2 × 10^9^ Pa and 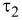 = 3 × 10^−8^ s. (c) Storage and loss modulus for material 1, plotted in a wider frequency range than in Figure 2.

Finally, it is worth considering the expected viscoelastic nature of the material, if such information is available. Although the set of Maxwell model parameters that leads to a specific behavior of a particular harmonic viscoelastic function (e.g., storage modulus, loss modulus, and loss angle) is not unique (multiple sets of parameters can give a similar frequency dependence), the user may have prior clues about the type of material being characterized, which can aid in the development of an appropriate viscoelastic model and in the selection of the characterization frequencies and experimental methods to be used (i.e., the type of cantilever and the type of experiment). For example, one may consider a hypothetical polymeric material that has multiple levels of structure, each leading to different characteristic times, as it is known that the molecular structure is directly related to the viscoelastic response [39–41]. One may imagine that molecule–molecule sliding (on the nanometer scale) may lead to a relatively fast characteristic time [42]. If the material forms superstructures (e.g., lamellae), these may give rise to an additional characteristic time scale, which may be larger than the molecular time scale due to the larger size of the superstructures compared to the molecules [43]. Furthermore, one may observe that the types and arrangement of the superstructures lead to even larger domains, which may be responsible for yet another characteristic time in the deformation of the material, and so on [39,43]. With this information, one may postulate a viscoelastic model that has characteristic times (i.e., Maxwell arms) matching those expected from considering the physical behavior of the system. While this by itself does not suffice in fully solving the property inversion problem for dynamic AFM, it provides important insight for future approaches of doing so. This could, for example, be important in the case of two materials that are identical in their viscoelastic properties in a certain frequency range, but differ in a wider range. For example, Figure 6a and Figure 6b compare the storage and loss modulus, respectively, of material 2 and a third material, which has the same viscoelastic elements of the model used to represent material 2 (i.e., the same Generalized Maxwell model parameters), but has one additional spring–damper arm added to it, which only responds at higher frequencies than those used above to characterize material 2 (70 and 350 kHz). As the graphs show, the parameters of these two materials are nearly identical in the previously considered frequency range, but differ very significantly at higher frequencies, say 2 or 3 MHz. Similarly, the moduli calculated for material 1 in the range of frequencies that is relevant to material 2 in Figure 2, appear to only vary slowly with frequency, but this is because we had not considered the full range of frequencies that is relevant to material 1. Figure 6c shows an expanded frequency representation, where this type of viscoelastic behavior of the material is evident. One may not be able to fully explore the relevant frequency ranges with traditional AFM instruments, which do not generally have the required bandwidth (tens of megahertz in this case), but would at least be able to begin probing the corresponding types of behavior, say with multifrequency AFM [5,38,44] using a higher (i.e., the 3rd or 4th) eigenmode of a traditional tapping-mode cantilever at a frequency of about 5–10 MHz (or higher with an instrument that has a higher bandwidth). Exploiting the time–temperature superposition principle could also be useful in some cases to access a larger frequency range using currently available capabilities. Increasing the temperature of the sample can accelerate the motion of the structures and their relaxation times, so it is in principle possible to access short time scales (high frequencies) even with conventional experimental setups. However, this principle only applies to thermo-rheologically simple materials, such as certain types of simple polymers [16,18,45]. There may also emerge experimental complexities related to cantilever or sample temperature control [46].

In closing this section we again acknowledge that the problem of inverting the frequency-dependent viscoelastic behavior of a material from dynamic AFM observables is a formidable task for which there does not yet exist a solution. However, we point out that there does exist a solution for quasi-static force–distance characterization, as described in the introduction [14,33–34], which can greatly inform the dynamic characterization, even if there still are some limitations in the frequency ranges that can be probed quasi-statically. Such a two-step approach can help to avoid some of the mischaracterization issues discussed above, especially in combination with numerical simulations of quasi-static [14,33–34] and dynamic AFM [35] measurements, which incorporate viscoelastic models such as those depicted in Figure 1.

## Conclusion

We have presented an analysis of the characterization of viscoelastic materials containing multiple characteristic times with intermittent-contact dynamic AFM, describing some of the intricacies in their mechanical response. Also, we especially cautioned the readers of a few common possibilities for mischaracterization that may emerge when probing such materials at only one frequency or at frequency ranges where the viscoelastic behavior of the material cannot be observed, or when using an inappropriate cantilever. In particular, we have shown that two very dissimilar materials may give nearly identical results, which would incorrectly lead to the conclusion that they are mechanically similar. While we recognize that the problem of inverting the frequency-dependent viscoelastic behavior of a material from dynamic AFM observables is a formidable task for which there does not yet exist a solution, there do exist solutions for quasi-static force–distance characterization [14,33–34], which can help to inform the dynamic characterization and thus help to mitigate the mischaracterization issues discussed in this manuscript. Finally, it is important to point out that our analysis is based on linear viscoelastic assumptions, which could be violated due to temperature gradients generated during tip–sample impact, phase changes of the material under the tip, nanometer-scale structural re-arrangements, and other phenomena, and which would result in nonlinear mechanical types of behavior, which could have strong effects on the AFM observables. We thus highly encourage future research on the incorporation of nonlinear viscoelastic analyses into AFM studies.

## Experimental

Simulation of the AFM cantilever was accomplished through a three-eigenmode vibratory model, where each eigenmode has a separate equation of motion, but all equations are coupled through the tip–sample force curve, as is customary in multifrequency AFM simulations [24,38,47–48] or in simulations of AFM imaging in liquid environments [49–50]. The higher eigenfrequencies have the customary rectangular-beam relationship to the fundamental eigenfrequency [5], but only the fundamental eigenfrequency is driven. The cantilever parameters used are provided in Table 2. The tip–sample forces consist of two components. The attractive van der Waals interactions were modeled through the Hamaker equation, as is also customary in AFM [2]. The repulsive interactions were represented using Generalized Maxwell models (Figure 1b) with the parameters given in Table 1. The AFM tip was treated as a spherical indenter with a radius of curvature of 5 nm, and its incorporation into the viscoelastic model followed the numerical implementation of the method of dimensionality reduction (MDR) [51]. The four coupled equations (three cantilever eigenmodes and the viscoelastic model relaxation and force calculation) were integrated numerically. The simulation procedures have been discussed in detail in previous publications and their supporting information files [14,33,35].

**Table 2:** AFM simulation parameters.

cantilever fundamental resonance frequency, ν_1_, kHz	70 or 350
cantilever force constant, N/m	0.5 or 5
quality factors for first three eigenmodes: *Q*_1_, *Q*_2_, *Q*_3_ (all simulations)	150, 300, 450
tip radius of curvature, nm	5
Hamaker constant, J	7.1 × 10^−20^
fundamental eigenmode free oscillation amplitude, nm	50
